# Effects of the Covid-19 pandemic on maternity staff in 2020 – a scoping review

**DOI:** 10.1186/s12913-021-07377-1

**Published:** 2021-12-27

**Authors:** Nadine Schmitt, Elke Mattern, Eva Cignacco, Gregor Seliger, Martina König-Bachmann, Sabine Striebich, Gertrud M. Ayerle

**Affiliations:** 1 Institute of Health and Nursing Science, Center for Health Sciences, Medical Faculty of Martin Luther University Halle-Wittenberg, Magdeburger Straße 8, 06112 Halle, Saale Germany; 2 Department of Applied Health Sciences, University of Applied Sciences Bochum, Bochum, Germany; 3 Department of Health Professions, Bern University of Applied Sciences, Bern, Switzerland; 4 Department of Obstetrics and Prenatal Medicine, Center of Fetal Surgery, University Hospital Halle (Saale) and Center for Reproductive Medicine and Andrology, Martin Luther University Halle-Wittenberg, Saale, Halle, Germany; 5 Health University of Applied Sciences Tyrol, Innsbruck, Austria

**Keywords:** Scoping review, maternity staff, Covid-19 pandemic, obstetrician, midwife

## Abstract

In the spring of 2020, the SARS-CoV-2 virus caused the Covid-19 pandemic, bringing with it drastic changes and challenges for health systems and medical staff. Among the affected were obstetricians and midwives, whose close physical contact with pregnant women, women who recently gave birth, and their children was indispensable. In the obstetric setting, births cannot be postponed, and maternity staff had to adapt to assure obstetric safety while balancing evidence-based standards with the new challenges posed by the pandemic. This scoping review gives a comprehensive overview of the effecs the Covid-19 pandemic had on maternity staff. We followed the evidence-based approach described by Arksey & O’Malley: we searched several databases for English and German articles published between January 2020 and January 2021 that discussed or touched upon the effects the pandemic had on maternity staff in OECD countries and China. We found that structural challenges caused by the crisis and its subjective effects on maternity staff fell into two main topic areas. Structural challenges (the first main topic) were divided into five subtopics: staff shortages and restructuring; personal protective equipment and tests; switching to virtual communication; handling women with a positive SARS-CoV-2 infection; and excluding accompanying persons. The pandemic also strongly affected the staff’s mental health (the second main topic.) Attempting to meet challenges posed by the pandemic while afraid of contamination, suffering overwork and exhaustion, and struggling to resolve ethical-moral dilemmas had severe negative subjective effects. Several studies indicated increased depression, anxiety, stress levels, and risk of post-traumatic stress symptoms, although the crisis also generated strong occupational solidarity. Care for pregnant, birthing, and breast-feeding women cannot be interrupted, even during a pandemic crisis that requires social distancing. Maternity staff sometimes had to abandon normal standards of obstetric care and were confronted with enormous challenges and structural adjustments that did not leave them unscathed: their mental health suffered considerably. Researchers should study maternity staff’s experiences during the pandemic to prepare recommendations that will protect staff during future epidemics.

**Supplementary Information:**

The online version contains supplementary material available at 10.1186/s12913-021-07377-1.

## Background

Obstetric care in 2020 was dominated by the Covid-19 pandemic, in which suddenly increasing numbers of infections and deaths threatened to break down healthcare systems across the world as they struggled to introduce necessary measures to protect the population from infection. Along with normal hygiene measures like disinfection and wearing protective clothing, more drastic measures, designed to prevent or slow contamination, included isolating patients with Covid-19, tracking and contacting those infected or exposed, and enforcing social distancing [1].

Staff in health care made large adjustments to the health care delivery system to prevent infection [2, 3]. Thus, the pandemic posed enormous challenges and required massive change to care also to maternity staff; prenatal examinations and births could not be postponed, unlike routine medical procedures [4, 5]. At least 116 million babies will be born during the pandemic and its aftermath; millions of women will need care during pregnancy, delivery, and childbed [6]. UNICEF underlines the pressing need for healthcare personnel to ensure women can continue to access healthcare services [6]. Maternity staff are usually in close physical contact with pregnant women and women giving birth and thus were at high risk of infection [7], especially since infected pregnant women often are asymptomatic or have moderate Covid-19 infections that are hard to detect [8–11]. In a Portuguese study, eighty-two percent of the cases of pregnant women had no symptoms [9], this is about the average of asymptomatic cases in the general population.

Great uncertainty, massive restrictions in the daily work lives of maternity staff, and other challenges posed by the crisis have are likely to have strongly affected maternity staff [12]. Since the mental health and psychosocial well-being of medical staff during the pandemic is as important as their physical health [13], we need to understand both the structural and organizational effects of the Covid-19 pandemic and its psychological effects on maternity staff. We aimed to deepen our insights with a scoping review that could serve as a foundation for future research.

The aim of our scoping review is to summarize literature on the effects of the Covid-19 pandemic on maternity staff in the year 2020 and to describe the present state of research on this topic. On the basis of the published literature we would like to make recommendations for future research projects.

## Methods

To conduct the scoping review process in a rigorous and transparent way, Arksey & O’Malley provide a framework consisting of five stages. These stages are: identifying the research question, identifying relevant studies, study selection, charting the data and collating, summarizing and reporting the results [14]. In the following paragraphs we followed this framework and divided this scoping review into the stages defined by Arksey & O’Malley in order to map the existing literature on the impact of the Covid-19 pandemic on maternity staff.

Our literature search and subsequent review followed PRISMA guidelines (Preferred Reporting Items for Systematic Reviews and Meta-analysis Protocols) [15] and we also applied the PRISMA extension for scoping reviews [16] (See Additional File 1). The PRISMA extension for scoping reviews is a checklist with 20 reporting items and 2 optional items that we consider when conducting our scoping review. Every item includes a declaration and an example of good reporting. We used this checklist to ensure that we have considered all the major elements of a scoping review.

### Identifying the research question

What publications on the experiences of maternity staff in the OECD countries and China during the Covid-19 pandemic were published in 2020 and early 2021 and what impact of the pandemic on maternity staff are reported in these publications?

### Identifying relevant studies

From December 2020 to February 2021, we searched the CINAHL, MEDLINE (via Ovid, Web of Science), Cochrane Library, and PubMed databases. We searched titles and abstracts, based on the search string (midwi* OR nurse-midwi* OR certified midwi* OR obstetric nurses OR obstetric* OR perinatal care OR maternity care) AND (burden OR workload OR barriers OR challenges OR safety OR stress OR mental health OR resources OR potential OR anxi* OR depression OR psych*) AND (covid OR pandemic OR coronavirus). We tailored the string to each database’s search syntax (See Additional File 2).

We also made use of free and bespoke literature search packs provided by MIDIRS and manually searched the German Midwives Journal (Deutsche Hebammenzeitschrift [DHZ]), the Midwives’ Forum (Hebammenforum) of the German Midwives Association, the Journal of Midwifery Science (Zeitschrift für Hebammenwissenschaft) of the German Society of Midwifery Science, and The Midwife (Die Hebamme). To identify more publications, we asked colleagues for their recommendations. We also checked the references of our publications to identify references we might have missed.

### Study selection

Two independent reviewers (NS and AR) scanned all the titles and abstracts (or full text if title and abstract were not available) and excluded publications based on our criteria. They resolved discrepancies through consensus, inviting comments from a third reviewer (GA) if necessary. Then two independent researchers (NS and EM) read the full text of the remaining publications and included those that met our criteria. We did not appraise methodological quality or risk of bias of the included articles, which is consistent with guidance on scoping review conduct [3].

We included publications that discussed the effects of the Covid-19 pandemic on maternity staff in 2020: scientific studies; case reports; reports, editorials; letters to the editor; interviews; commentaries; and, newspaper articles with quantifiable evidence. Among these were non-peer-reviewed texts by experts. We considered all publications in German and English published between January 2020 and January 2021. Our definition of maternity staff included midwives, obstetricians, obstetric nurses, and nurse-midwives.

We included publications from China because it was the initial site of the outbreak. We excluded publications that did not originate in China or the OECD countries. The different health care systems of different countries are very difficult to compare with each other. Since it is even more difficult to compare the obstetric system of an emerging or developing country with that of an industrialized nation, we decided to focus exclusively on countries that are members of the OECD.

We also excluded publications that focused on the effects of training or continuing education for midwives and obstetricians, along with purely informative recommendations, speculations, and guidelines. We excluded general news items without quantifiable evidence and reports by medical staff not working in obstetrics. We also excluded publications that focused on the effects of the pandemic on pregnant women, women giving birth, and women in childbed because they were not directly related to the effects of the pandemic on staff.

Many of titles we identified discussed the switch from face-to-face to alternative forms of communication, especially digital, but we included only publications that discussed the implementation of these alternative methods and the challenges they posed to maternity staff. We excluded publications that described digital systems and care models in detail, further development of such systems, or patient satisfaction.

After reading the full text two authors (NS and EM) organized the literature topically. They discussed the topics that reflected the impact of the Covid-19 pandemic on maternity staff and allocated the literature to the topics.

### Chart the data

To prepare the descriptive summary, we created an analytical framework for reading the publications, and created a table following Arksey & O’Malley’s design, into which we extracted source data [14] and can be seen in Table 1.

**Table 1:** Studies included in the scoping review

No.	Author, Year	Title	Type of Article	Topic	Country of focus	Participants	Conclusion
1	Aksoy & Koçak, July 2020	Psychological effects of nurses and midwives due to COVID-19 outbreak: The case of Turkey	Cross-sectional study peer reviewed	Mental health	Turkey	758 nurses and midwives	The respondents were afraid, especially of infecting their relatives, and were unsure how to deal with each other. But they are also proud of their work.
2	Alfieri et al., November 2020	COVID-19 does not stop obstetrics: what we need to change to go on safely birthing. The experience of a University Obstetrics and Gynecology Department in Milan	Commentary Peer reviewed	Structural changes	Italy	/	To ensure continual care and safety of mothers and newborns, organizational changes were made within the maternity ward.
3	Aust, April 2020	Corona, der erste April - und was das mit uns macht [Corona, the first April - and what it did to us]	Non-scientific interview	Personal experiences Staff shortage and restructuring Personal Protective Equipment and tests Switch to virtual communication	Germany	Midwife in independent practices	Despite changes in working conditions, and lack of protective equipment and disinfectant, there was a wave of solidarity and mutual support.
4	Aziz et al., July 2020	Adaptation of prenatal care and ultrasound	Report Peer reviewed	Personal Protective Equipment and tests Switch to virtual communication Dealing with infected women	USA	A maternity hospital with annually ca. 4600 pregnant women	The intervals between prenatal examinations and the number of ultrasonic sounds changed. Some necessary interventions for genetical diagnostics and fetal therapy were postponed and some appointments were switched to telehealth.
5	Aziz et al., May 2020	Telehealth for High-Risk Pregnancies in the Setting of the COVID-19 Pandemic	Description Peer reviewed	Switch to virtual communication	USA	Hospital group in New York with 2 clinics and 6900 births	The number and implementation of prenatal care examinations were changed and telehealth for high-risk pregnancies were described in detail.
6	Bahat et al., August 2020	Evaluating the effects of the COVID-19 pandemic on the physical and mental well-being of obstetricians and gynecologists in Turkey	Cross-sectional study Peer reviewed	Mental health	Turkey	253 obstetricians and gynecologists	Many respondents were anxious about contact with infected pregnant women, reported feeling desperate, and isolated themselves from their families. At the same time, their perception of their work changed for the better and they had the feeling were able to take adequate care of mothers and their newborns.
7	Bailey & Nightingale, June 2020	Navigating maternity service redesign in a global pandemic: A report from the field	Report Peer reviewed	Staff shortage and restructuring Personal Protective Equipment and tests Switch to virtual communication Dealing with infected women Exclusion of accompanying persons	UK	A large teaching hospital with 6000 births	Report on pandemic-related staffing and spatial changes of obstetric teams and necessary adjustments to meet individual and governmental requirements.
8	Baumann et al., December 2020	Adaptation of independent midwives to the COVID-19 pandemic: a national descriptive survey	Cross-sectional study Peer reviewed	Personal Protective Equipment and tests Switch to virtual communication	France	1517 midwives in independent practice, 20% of all midwives in independent practice in France	91% of these midwives changed the services they offered. They cancelled home visits and course meetings and increased phone calls. Some closed their practices.
9	Baumgarten, June 2020	Hebammen sind systemrelevant [Midwives are relevant to the system]	Non-scientific interview	Staff shortage and restructuring Personal Protective Equipment and tests Switch to virtual communication Exclusion of accompanying persons	Germany	Advisory Council of the German Association of Midwives	In some federal states in Germany, independent midwives were not initially included in essential professions. They received no protective clothing, lost a large portion of their earnings, and had no designated contact person.
10	Becker, June 2020	„Die Coronakrise zeigt uns die Lücken im System“ ["The Corona crisis shows us the gaps in the system"]	Non-scientific interview	Personal experiences	Germany	2 midwives and the president of the German Association of Midwives	Maternity staff had to cope with changes in their daily professional life and with their own worries. But they also described positive effects.
11	Bender et al., July 2020	The Psychological Experience of Obstetric Patients and Health Care Workers after Implementation of Universal SARS-CoV-2 Testing	Cross-sectional study Peer reviewed	Mental health	USA	158 persons from maternity staff	The staff's anxiety and job satisfaction were worse than before the pandemic, but routine testing showed positive effects. Staff also worried about the increased distress of mothers who were separated from their newborns.
12	Campbell et al., November 2020	Consolidation of obstetric services in a public health emergency	Report Peer reviewed	Staff shortage and restructuring Personal Protective Equipment and tests Switch to virtual communication Dealing with infected women	USA	Two affiliated obstetric units in New York	Reports from three clinics about changes and new procedures to prevent infection, quick discharges, online triage, and staff shortage. Lack of equipment and new teams sparked fears that should be met with transparency to make the staff feel safer.
13	Chervenak et al., April 2020	Expanding the concept of the professional integrity of obstetrics during a public health emergency	Report Peer reviewed	Staff shortage and restructuring	USA	A clinic in New York	The pandemic changed medical care targets and needs. Individual patient welfare was made secondary to preventing mortality and protecting the whole population.
14	Corbett et al., August 2020	Anxiety and depression scores in maternity healthcare workers during the Covid-19 pandemic	Cross-sectional study Peer reviewed	Mental health	Ireland	240 maternity healthcare workers (midwives/nurses, obstetricians, laboratory staff, clerical/administrative staff, support staff)	About a fifth of those interviewed had moderate to severe anxiety and depression scores. Younger, female administrative personnel were more severely affected.
15	Coxon et al., June 2020	The impact of the coronavirus (COVID-19) pandemic on maternity care in Europe	Editorial	Staff shortage and restructuring Personal Protective Equipment and tests Switch to virtual communication Dealing with infected women Exclusion of accompanying persons	Europe	Practices in Europe	European countries maintained antenatal care differently during the pandemic. Some birthing centers closed down because emergency services had no more capacities; others were kept open so women do not have to go to the clinic.
16	Danvers & Dolan, July 2020	Women's Health During the COVID-19 Surge in the Bronx: Reflections from Two OBGYNs on the Flatter	Commentary Peer reviewed	Personal experiences Switch to virtual communication	USA	/	Maternity wards were reorganized, but were still familiar to staff. They spoke of longer work days, anxiety and isolation, and also bonding between staff and, during virtual meetings, with women as well.
17	Davis-Floyd et al., July 2020	Pregnancy, Birth and the COVID-19 Pandemic in the United States	Cross-sectional study Peer reviewed	Staff shortage and restructuring Personal Protective Equipment and tests Switch to virtual communication Dealing with infected women Exclusion of accompanying persons	USA	Maternity staff, 41 responses	The Covid-19 pandemic could change the responsibilities of certified nurse-midwives (CNMs) and certified professional midwives (CPMs) in the USA.
18	Dethier & Abernathy, June 2020	Maintaining certainty in the most uncertain of times	Commentary Peer reviewed	Personal experiences	USA	Staff of the department of obstetrics and gynecology in 2 hospitals in Boston	The authors speak of a new reality in which one had to work against one's beliefs, wrapped in protective clothing, without any physical contact.
19	Dunne, September 2020	Two in three members recovered from Covid-19 hit by post-viral fatigue	Newspaper article (Report on survey)	Mental health, physical effects	Ireland	7068 nurses and midwives	Most respondents complained about effects on mental health. Personnel who had trouble procuring protective clothing were twice as likely to be infected. Infected personnel complained mainly of exhaustion. Their greatest fear was infecting members of their household.
20	Furuta, August 2020	2020 International Year of Midwifery - In the midst of a pandemic	Editorial Peer reviewed	Switch to virtual communication Dealing with infected women Exclusion of accompanying persons	Japan	/	Maternity staff cared for women who could not give birth as usual, with extended families present, because of travel restrictions.
21	González-Timoneda et al., December 2020	Experiences and attitudes of midwives during the birth of a pregnant woman with COVID-19 infection: A qualitative study	Qualitative study Peer reviewed	Mental health	Spain	14 midwives	Midwives reported on several factors reduced their ability to provide a safe and respectful environment (higher work demands, supply of protective equipment, support from employers and colleagues, reliable guidelines). Midwives reported feelings of anxiety, agitation, insecurity and discomfort.
22	Green et al., July 2020	Providing women's health care during COVID-19: Personal and professional challenges faced by health workers	Editorial Peer reviewed	Exclusion of accompanying persons/women giving birth alone Personal protective equipment and tests Challenges due to less capacities (ethical dilemmas) Mental health	USA	/	Excluding accompanying persons put staff under more pressure to provide more emotional support. Medical staff could be victims of violence when they were seen as SARS-CoV-2 carriers.
23	Holton et al., October 2020	Psychological well-being of Australian hospital clinical staff during the COVID-19 pandemic	Cross-sectional study Peer reviewed	Mental health	Australia	668 hospital clinical staff (nurses, midwives, doctors and allied health staff)	Midwives and nurses had higher depression, anxiety, and stress scores than other groups in the health system. The scores were higher for those with less clinical experience, poorer health, and more concerns about Covid-19.
24	Horsch et al., June 2020	Moral and mental health challenges faced by maternity staff during the COVID-19 pandemic	Commentary	Mental health	Switzerland, Ireland, UK	/	Pandemic requirements that conflict with evidence, professional recommendations, and ethical and moral values may lead to professional moral impairment. Staff can feel like instruments of inhumane treatment and can become desensitized to preserve themselves.
25	Jeganathan et al., November 2020	Adherence and acceptability of telehealth appointments for high-risk obstetrical patients during the coronavirus disease 2019 pandemic	Longitudinal study Peer reviewed	Switch to virtual communication	USA	33 surveys of maternity staff	Positive balance for risk pregnancies via telehealth but half of providers want to return to face-to-face meetings when the pandemic is over.
26	Johnson et al., December 2020	COVID-19 Testing, Personal Protective Equipment, and Staffing Strategies Vary at Obstetrics Centers across the Country	Letter to the editor (report on surveys)	Personal Protective Equipment and tests	USA	Online survey at 315 obstetric centers	Different standards apply though infections increased. Use of N95 masks increased only slightly over time; use was not nationwide, possibly because of small budgets and poor access to protective equipment in municipal facilities.
27	Khot & Kumar, August 2020	Flattening the anxiety curve: Obstetricians' response to the COVID-19 pandemic in Victoria	Letter to the editor (small pilot study; semi-structured interviews)	Physicians' experiences	Australia	12 practitioners providing private maternity care	Physicians have many anxieties but also a strong feeling of belonging. Collegial relationships make it possible for them to cope with a rapidly changing situation and adapt to change.
28	Kiefer et al., December 2020	High frequency of posttraumatic stress symptoms among US obstetrical and gynecologic providers during the coronavirus disease 2019 pandemic	Cross-sectional study Peer reviewed	Mental health	USA	558 physicians, certified nurse midwives and nurse practitioners	Female gender, previous trauma, high perceived Covid-19 risk, and greater anxiety about Covid-19 increase the likelihood of posttraumatic stress symptoms in maternity staff.
29	Kumaraswami et al., September 2020	Response of an Obstetric Unit during the Coronavirus Disease of 2019 (COVID-19) Pandemic: Experiences from a Tertiary Care Center	Case report Peer reviewed	Structural changes	USA	/	To ensure staff and women's safety, health care workers devise different strategies and adjust clinical practice.
30	Lauer et al., October 2020	PPE during a pandemic: The experience of obtaining PPE and lessons learned from a department of obstetrics and gynecology in New York city	Report Peer reviewed	Personal Protective Equipment and tests	USA	/	There were reports of concern about obtaining respiratory masks when the number of Covid-19 positive women increased; there is still too little protective clothing. Competition for protective clothing and uncertainty about the pandemic lead possibly to unnecessary or too sparing use of protective clothing.
31	Liu et al., December 2020	Psychological impact in non-infectious disease specialists who had direct contact with patients with COVID-19	Cross-sectional study Peer reviewed	Mental health	China	2126 obstetricians and midwives	Higher risk of infection tracks higher depression and anxiety scores and sleep disturbance incidence. Protective equipment and training that prepares staff to deal with Covid-19 have a protective effect.
32	Madden et al., June 2020	Telehealth Uptake into Prenatal Care and Provider Attitudes during the COVID-19 Pandemic in New York City: A Quantitative and Qualitative Analysis	Quantitative and qualitative study Peer reviewed	Switch to virtual communication	USA	36 providers of prenatal care (mainly medical doctors)	Telehealth can be quickly implemented and is evaluated positively by the staff. Before the pandemic, less than half the staff wanted to use Telehealth. During the pandemic, nearly 90% of participants wanted to use it.
33	Murtada et al., July 2020	Managing an obstetrics and gynaecology department in time of COVID pandemic: safety and efficacy first at Foch hospital	Brief report Peer reviewed	Structural changes	France	/	Obstetrical care is adapted: important consultations are maintained, follow-up examinations are carried out via telehealth, and women with positive Covid-19 are treated separately. Lack of protective equipment at the start of the pandemic caused most infections in personnel.
34	Onwuzurike et al., June 2020	Examining Inequities Associated With Changes in Obstetric and Gynecologic Care Delivery During the Coronavirus Disease 2019 (COVID-19) Pandemic	Commentary	Switch to virtual communication	USA	/	Pandemic-related changes in the care of pregnant women discriminate against women of colour.
35	Peahl et al., October 2020	Patient and provider perspectives of a new prenatal care model introduced in response to the coronavirus disease 2019 pandemic	Modelling evaluation Peer reviewed	Switch to virtual communication Dealing with infected women	USA	A small town clinic with 150 maternity care providers (including 63 resident physicians)	Weekly prenatal examinations are cut by 16.1%, virtual meetings increase 32.5%; 53.3% of women and 62.1% of professional carers confirm virtual meetings do not endanger the safety of mother and child.
36	Peña et al., May 2020	A Survey of Labor and Delivery Practices in New York City during the COVID-19 Pandemic	Cross-sectional study Peer reviewed	Personal Protective Equipment and tests Dealing with infected women Exclusion of accompanying persons	USA	Senior consultants in 4 urban hospitals in New York	At first, accompanying persons were not allowed. Later, a screened or tested person was allowed to be present in the delivery room. Staff recommended women have a PDA so they would not require full anesthetic for a section in an emergency. Rooming-in and breastfeeding were always possible. Women were discharged as quickly as possible and continued to receive care at home via telephone.
37	Perrine et al., November 2020	Implementation of Hospital Practices Supportive of Breastfeeding in the Context of COVID-19 - United States, July 15-August 20, 2020	Cross-sectional study	Dealing with infected women	USA	1344 hospitals	In case of possible Covid-19 infection, skin contact was discouraged in 14% of clinics surveyed and prohibited in 16.5%. Rooming-in was discouraged in 37.8% of the clinics if an infection was suspected or diagnosed and prohibited in 5.3%. Women received less support for breastfeeding and were discharged more quickly.
38	Pietrasanta et al., May 2020	Management of the mother-infant dyad with suspected or confirmed SARS-CoV-2 infection in a highly epidemic context	Report Peer reviewed	Structural changes Personal Protective Equipment and tests Dealing with infected women Exclusion of accompanying persons	Italy	5 hub centers designated to centralise all cases of infected pregnant mothers	Hospital management faced challenges and reacted by restructuring several maternity wards.
39	Pluym et al., September 2020	Obstetrical Unit Response to the COVID-19 Pandemic: OUR Study	Cross-sectional study Peer reviewed	Personal Protective Equipment and tests	USA	Obstetrical unit response	A survey in 301 clinics in 48 US states found inadequate protective clothing and testing capacity. Municipal clinics in particular remained inadequately equipped over time.
40	Reforma et al., November 2020	A multidisciplinary telemedicine model for management of coronavirus disease 2019 (COVID-19) in obstetrical patients	Implementation study Peer reviewed	Switch to virtual communication Dealing with infected women	USA	Prenatal and postnatal care in 3 community centers with 5 satellite offices and 3 practices	A multidisciplinary telemedicine surveillance model was adopted to care for women who might be infected with Covid-19 during pregnancy and after birth. Video-calls might be used to recruit women who would otherwise not be reached (e.g., because the journey was too long, or if they didn't have time or lacked childcare).
41	Rochelson et al., May 2020	The care of pregnant women during the COVID-19 pandemic – response of a large health system in metropolitan New York	Report Peer reviewed	Staff shortage and restructuring Personal Protective Equipment and tests Switch to virtual communication Dealing with infected women Exclusion of accompanying persons	USA	10 large obstetric departments in New York with an annual total of 30,000 births	Systemic changes in the care procedures for pregnant women, women giving birth, and postpartum women reduced direct contact and number of examinations and increased e-health.
42	Saiman et al., November 2020	Infection prevention and control for labor and delivery, well baby nurseries, and neonatal intensive care units	Report Peer reviewed	Structural changes Personal Protective Equipment and tests Switch to virtual communication Dealing with infected women Exclusion of accompanying persons	USA	/	Various restructuring measures were taken to prevent infections in staff, patients, and accompanying persons. Guidelines and procedures have been developed for obstetric staff.
43	Semaan et al., June 2020	Voices from the frontline: findings from a thematic analysis of a rapid online global survey of maternal and newborn health professionals facing the COVID-19 pandemic	Cross-sectional study Peer reviewed	Structural changes Mental health Personal Protective Equipment and tests Switch to virtual communication Dealing with infected women Exclusion of accompanying persons	Worldwide	714 persons from maternity staff	Findings on subjective effects show higher stress, greater workload due to staff shortages, frequent changes in schedules, and exhaustion. The experiences of various professional groups that come into contact with potentially infected pregnant women and postpartum women were documented.
44	Shah et al., August 2020	Mental health amongst obstetrics and gynaecology doctors during the COVID-19 pandemic: Results of a UK-wide study	Cross-sectional study Peer reviewed	Mental health	UK	207 obstetricians and gynaecologists	During the pandemic, obstetricians and gynaecologists had worse mental health than the general public because they had to cope with continually changing guidelines, the pandemic, and their fear of infection.
45	Sögüt et al., June 2020	The relationship between COVID-19 knowledge levels and anxiety states of midwifery students during the outbreak: A cross-sectional web-based survey	Cross-sectional study Peer reviewed	Mental health	Turkey	972 midwifery students	No connection was found between anxiety and knowledge about Covid-19. Anxiety scores were higher for midwifery students whose parents were chronically ill or who were deployed in clinics again after the lockdown.
46	Steppat, May 2020	Blitzlichter aus dem Klinikalltag in Corona-Zeiten [Flashes from everyday hospital life in Corona times]	Report	Exclusion of accompanying persons	Germany	Maternity staff from several hospitals	The situation is characterized by constant innovation and staff waiting for Covid-19 positive women. Prenatal classes were suspended. Although women were more often alone in the delivery room and there was more peace and quiet, they did not receive 1:1 care.
47	Teubner, August 2020	Veränderungen im Hebammenalltag durch die Corona-Krise [Changes in the everyday life of midwives during the corona crisis]	Report	Switch to virtual communication Exclusion of accompanying persons	Germany	/	Video-calls were perceived as a good alternative. The maternity ward is quieter and more peaceful and breastfeeding as needed was more acceptable.
48	Uytenbogaardt, June 2020	COVID-19's effect on midwives' mental health	Editorial	Mental health	UK	4036 midwives and nurses	The ICON study (Impact of COVID-19 on the Nursing and Midwifery workforce) found that the greatest fear of the staff was of infecting family members. Only 1% of midwives used the online mental health forum provided by the National Health System.
49	Uzun et al., May 2020	Psychological and social effects of COVID-19 pandemic on obstetrics and gynecology employees	Cross-sectional study Peer reviewed	Mental health	Turkey	13 doctors, 52 midwives and 38 nurses	There were no significant mental health differences between age groups or genders across the three professions (doctors, midwives, nurses).
50	Vierlinger et al., June 2020	Have you got any (digital) solutions on how to best reach women and families under Covid-19?	Report	Switch to virtual communication	Germany	5 midwives	Online events reached women who would not have been able to attend face-to-face sessions (e.g., due to preterm labor, childcare). Midwives wre proud that they could offer videos and online courses.
51	Wegrzynowska et al., October 2020	Polish maternity services in times of crisis: in search of quality care for pregnant women and their babies	Secondary analysis and expert interviews Peer reviewed	Switch to virtual communication Exclusion of accompanying persons	Poland	6 healthcare professionals including midwives and midwives in management positions; 1 pregnant woman	Prenatal care was organized outside the clinic to protect against infection but births took place in the clinic as usual.
52	Wilson et al., June 2020	Caring for the carers: Ensuring the provision of quality maternity care during a global pandemic	Report Peer reviewed (ahead of print)	Mental health	Australia	/	The workload increased in the pandemic. Stress factors included the need to adhere to constantly changing guidelines, reassure patients and family members, cope with their own worries, and continue working normally.
53	Yates et al., July 2020	The Response to a Pandemic at Columbia University Irving Medical Center's Department of Obstetrics and Gynecology	Report Peer reviewed	Staff shortage and restructuring Personal Protective Equipment and tests Switch to virtual communication Dealing with infected women Exclusion of accompanying persons	USA	A gynecological hospital in New York	Daily virtual meetings of clinic employees created solidarity and a feeling of fighting Covid-19 together as a team. Contact with women shifted to video-calls.
54	Yörük & Güler, October 2020	The relationship between psychological resilience, burnout, stress, and sociodemographic factors with depression in nurses and midwives during the COVID-19 pandemic: A cross-sectional study in Turkey	Cross-sectional study Peer reviewed	Mental health	Turkey	377 nurses and midwives	A third of midwives suffered depression caused by higher stress levels and emotional exhaustion. A high resilience score had a protective effect. Risk of depression was higher in midwives than in nurses, by a factor of 1.92.

The search through the data bases returned 889 published publications from the period between January 2020 and February 2021. After de-duplicating, 346 publications remained. We added 63 unique publications to that number through hand searches, inquiries to colleagues, and reference checking, raising the total of unique publications to 409. After our reviewers searched through their titles and abstracts, they excluded 330. After first review of the remaining 79 publications, we identified two main topics. The first was structural challenges posed by the pandemic and the adjustments maternity staff made to adapt to new circumstances. The second was subjective effects of the pandemic on the staff, particularly psychological effects.

From the 79 publications, we excluded another 25 after reviewing the full texts. We excluded those that only made recommendations based on studies of previous crises (SARS, H1N1/09 etc.), recommendations and advice from maternity staff that did not focus on the staff’s individual problems or their mental health issues, or the challenges the Covid-19 pandemic posed, and those that proposed general guidelines or recommendations for care of pregnant women and women in childbed, but did not focus specifically on maternity staff. We also excluded three publications because we could not locate the full texts.

**Fig. 1:**
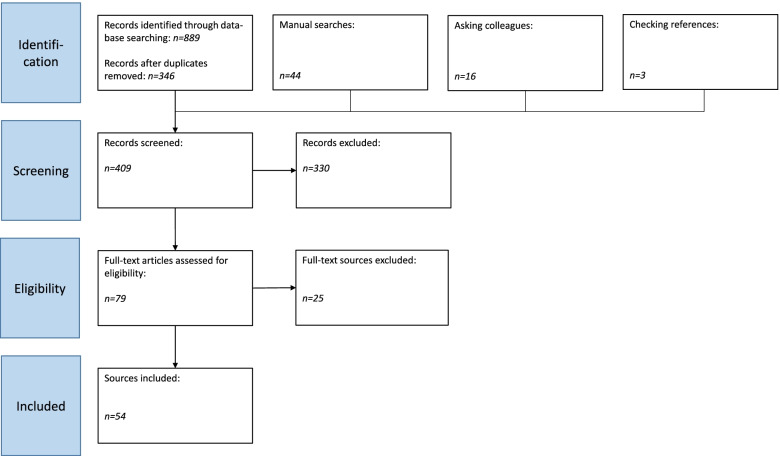
Flow Chart of literature selection

We included the remaining 54 publications. For a flow chart of publication selection, see Fig. 1.

Of the 54 publications included, 40 were peer-reviewed articles and 14 had not been peer-reviewed. They comprised mainly scientific study results, and also case reports, reports, editorials, letters to the editor, interviews, commentaries, and one newspaper article. The first article was published in April 2020 and the last in December 2020; 16 publications were from the European Union, 24 from the USA, five from Turkey, three each from the UK and Australia, and one each from Japan and China. One study was global.

### Collate, summarize, and report the results

One of the first authors (EM) wrote the narrative description of the first main topic we identified: structural and organizational challenges. The other first author (NS) wrote the narrative description of the second main topic: the subjective effect of the crisis. All authors checked each description for clarity and readability. All authors helped edit the descriptions for readability and accuracy.

## Main text/Results

**Fig. 2:**
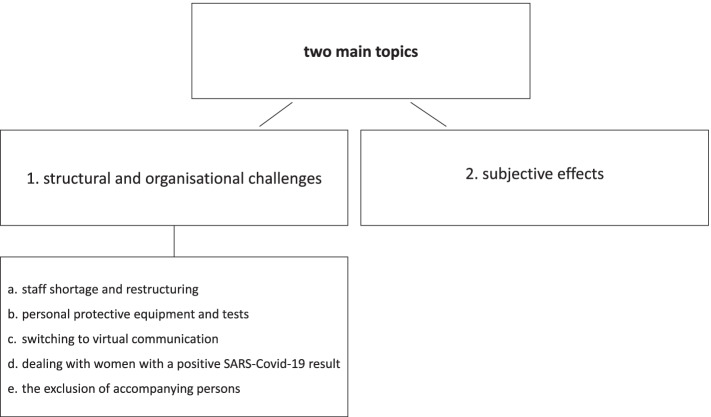
Overview of the topics

Our definition of the two main topics was reconfirmed as we continued our review. For the first topic (the structural and organizational challenges posed by the pandemic and adjustments made by maternity staff), we defined five subtopics: a) staff shortage and restructuring; b) personal protective equipment and tests; c) switching to virtual communication; d) dealing with maternity patients who tested positive for SARS-CoV-2; and, e) excluding accompanying persons. For the second topic, we described the subjective effects of the crisis on the mental health of maternity staff. For an overview of the main topics and the subtopics, see Fig. 2.

### Main topic: Structural and organizational challenges

#### Staff shortage and restructuring

After Covid-19 was declared a pandemic, lockdown in most countries soon led to staff shortages on obstetrical wards. Maternity staff with parental duties had to coordinate the care for their children when schools and kindergartens closed [5, 17–19]. Hospitals needed to arrange flexible duty rosters [20]. In New York, a physician described her attempt to balance her need to protect her own family against the needs of pregnant women who required continuous support at births, abortion appointments, prenatal examinations, and during medical treatment [19]. In Germany, midwives were not initially included in essential professions, so they were not provided with emergency childcare [17]. Maternity staff with underlying conditions and pregnant professional staff had to undergo a risk assessment before they could go back to work [5, 20]. Infected staff and staff in quarantine made the staff shortage in the UK worse. After the national call for self-isolation in the UK in March 2020, staff dropped out when they were infected and also as a precautionary measure after they came in contact with a Covid-19 patient [20]. In New York, a general 14-day quarantine was imposed on all staff members who spent longer than 10 minutes within 2 meters of a Covid-19-positive patient [21]. This strict regulation was later mitigated after wearing protective masks was required [21].

In New York there were reports that the health system would be massively restructured during the pandemic [5]. Maternity staff was assembled into new teams and they needed new instructions to make it easier to work together under pressure, placing high demands on maternity staff to be adaptive and flexible [5]. In Germany, to address the shortage, the German Midwives Association linked its website to an internet platform connecting voluntary helpers with hospitals [17]. Across Europe, retired staff were called back, school and university students were contracted for paid internships [18, 20] and, in the public health system, the hiring dates for newly-qualified obstetricians were advanced (especially for office work and organizational tasks) [18].

The approach was inconsistent across countries. In some countries, maternity hospitals were closed because emergency services lacked capacity for transfers. In others, maternity hospitals were kept open so pregnant women did not have to go to general hospitals [22]. The Netherlands made an official recommendation to give birth at home to reduce the number of people present at the birth [18]. In New York, an obstetrical ward was moved to a building far from the main building as a precaution [23], even though it took more time for consultants to get there and sometimes women had to be moved to the main building [23].

Cohorts formed in delivery rooms, while teams working on the wards or with outpatients were separated [19, 24]. Waiting areas were reorganized to reduce the risk of infecting patients and staff [4, 25]. In France, a gynecological and obstetrics area was turned into a Covid-19 ward [4] and in Italy maternity wards were designated as centers to which infected (or presumed infected) pregnant women must be admitted [26]. In many places, women who were required to go to hospital for examination or women who presented themselves at hospital were usually checked, via questionnaires or email, for potential symptoms before they entered the hospital [4, 21, 24, 26, 27]. There was a shortage of protective clothing and in a pandemic wave up to 11-26% of healthcare staff in European countries tested positive for Covid-19. Midwives were among the dead in the UK and Italy [18].

#### Personal protective equipment and tests

A complete set of protective clothing against contamination with Sars-CoV-2 consists of a respiratory mask (FFP2 or N95), a protective overall with hood, gloves, and protective goggles [5, 20, 26, 28–31]. Even in Europe protective clothing was not available everywhere [5, 25, 32]. Hospital wards treating Covid-19 positive patients were prioritized but initially delivery rooms were not [33]. By mid-March 2020, only 74.9% of outpatient midwives in France (n=1,136) had masks, 61.6% of these midwives had hand sanitizers for the patients, 15.6% had protective overalls, and 7.8% had goggles [34]. The lack of protective clothing made maternity staff and patients feel insecure, creating uncertainty and fear of infection [20, 33]. Midwives want to use protective clothing responsibly, and to know that they are taking care of themselves and the women in their charge, in the hospital and during home visits [20].

Protective clothing became available very late for midwives in Germany [28] and the Netherlands who were not attached to a hospital [5, 18]. Protective clothing was not always distributed to them and sometimes they had to procure it themselves [17]. Midwives working outside hospitals received no standardized instructions [28] and did not have clear responsibilities [17].

Fear and worry also inspired support, help, and solidarity. Midwives spoke of receiving masks as gifts from nail design studios and veterinary practices [5] or of being given disinfectant by a company that could spare it [28, 32]. By mid-June 2020 in France, midwives working outside hospitals were eligible for six masks a week [34]. Some hospitals developed effective methods of recycling protective material [35].

In the hospitals, (video) courses instructed staff about implementing hygiene rules and correctly using protective clothing [5]. They also developed simulation exercises for time-critical emergencies [24]. So-called “dofficers” were made responsible for ensuring staff adhered to the rules and hospitals installed mirrors so staff could check to see if protective clothing fitted properly [5, 35]. Protective clothing makes the environment safer for both hospital patients and maternity staff [21]. Although protective clothing greatly increased safety, gowning up was time-consuming and staff feared they would not be able to react quickly enough in an emergency [33].

Even though the number of positive Covid-19 women in hospitals increased, the USA did not set hygiene standards specifically for the pandemic and standards varied within the country [29]. Since protective clothing was hard to procure, respiratory mask use increased only slightly over time; they were not worn at every birth [5, 29, 31], perhaps because communal facility budgets were tight and the materials were hard to access [29]. Repeated changes and restrictions unsettled maternity staff, who were not sure if protective clothing would prevent them or their families and colleagues from contracting Covid-19 [5]. In some European countries the maternity staff had to work without protective clothing [5, 32]. In some maternity hospitals, a midwife was only allowed to wear a respiratory mask during the birth [33], and in other hospitals complete protective clothing was compulsory at every birth, for self-protection and to protect the newborn child [5, 21]. A survey of 301 hospitals in 48 US states revealed that only 33% required complete protective clothing at each vaginal birth of asymptomatic women and 38% at caesarean sections [31].

Midwives also found that protective clothing made personal contact with women more difficult because the masks and goggles did not allow facial expressions [33] and their charges could not recognize a “comforting smile.” Communication in the “new normal” had to be readjusted accordingly [20, 36].

Testing is another important protection measure that, at first, was done only for symptomatic female patients or those in contact with infected persons [26]. Later, many hospitals began testing every woman who visited the hospital [4, 5, 21, 24, 29, 30]. There were 1,344 maternity hospitals in the USA: 90.2% had adequate testing capacities; 84.3% tested all pregnant women [30]. Later on, rules about wearing protective clothing depended on the results of PCR tests and symptoms indicating an infection with Covid-19, and took into account the lack of protective clothing or the discomfort of staff who wore it [21, 35, 37]. Insufficient protective clothing and inadequate testing capacity posed particular challenges to communal hospitals [29, 31, 36].

Baumann indicated hygiene rules were generally not practicable for home visits [34], which encouraged the adoption of video calls/telemedicine because it eliminated infection risk [38].

#### Changing to virtual communication

Using online media prevents infections and reduces the need for protective clothing and Covid-19 tests [38, 39]. In large and small maternity hospitals, virtual meetings with pregnant women and women in childbed are increasing [24, 36, 40, 41].

The need to work from home when possible to avoid contacts spurred the development and improvement of platforms for virtual medical staff meetings. These platforms were widely accepted by maternity staff, who ideally received training to use it effectively [42]. As early as March 2020, hospitals in New York established procedures to schedule daily or weekly virtual staff meetings [21, 36]. Yates et al. described daily virtual meetings of 150-200 employees at a time [36]. Maternity staff used these platforms to share information and experiences with Covid-19 patients and update recommendations for action, and they also used them to discuss personal matters. These regular meetings were well accepted overall and created community feeling and raised team spirit [5, 36].

Hotlines and virtual support were set up to reach many women easily via video call [21, 36]. In the outpatient sector, home visits grew shorter and the time between visits lengthened [28, 39]. Maternity staff also contacted women via telephone [21, 25, 28] and preferred video calls [18–21, 33, 34, 37, 38, 40, 43]. Midwives thus kept in touch with the patients regularly, even if they lacked protective clothing and tests and felt they were able to reduce the anxiety and stress of women and their families [34]. To ensure women received proper care, maternity staff produced videos about preparing for birth and situations that might arise giving birth and streamed courses during video conferences [4, 37, 42, 44]. Midwives were greatly praised by women and were proud of their quick adjustment to new circumstances [41, 43, 44].

Mixing home visits and online advice made daily work much easier for freelance midwives [43]. A female gynecologist in New York spoke of the intimacy and connectedness she felt during video calls that took place in women’s homes [19]. Teubner suggested continuing to provide online advice in the future [43], although digital presence could never replace personal visits [17]. A New York study found that 73.8% of women wanted to continue meeting via video call after the pandemic but 56% of the maternity staff did not want to continue the video meetings, though women cancelled far fewer video consultations than they cancelled office visits before the pandemic [40]. Another New York study found 92% of respondents thought telehealth technologies could guarantee adequate care. Though only 45% of them had taken advantage of existing telehealth technologies before the pandemic, 89% wanted to continue to use the technology after the pandemic [45]. Virtual meetings also enabled maternity staff to care for women who would not have otherwise had contact with a midwife because of barriers like travel time or other time limitations, health restrictions, or lack of available childcare [40, 41, 44, 46]. In Germany, there have been reports that laws are changing medical billing options for digital care; midwives could not bill for this before the pandemic [17, 28].

#### Dealing with women with positive SARS-CoV-2 test results

Ideally, women with positive Covid-19 test results or symptoms and uncertain infection status [46] would not enter hospitals or would be limited to short stays [33]. But women who give birth or have to go to the clinic because of complications may be exposed to infected women, so clinics had to do some restructuring. In the hospitals, separate areas, some with low pressure rooms [24, 27], were set up for infected women [4, 5, 21, 25]. Wards were closed down and some obstetrical units were moved to other buildings [20]. It was necessary to balance the requirement to separate infected and uninfected patients and staff with the urgent need to free as many beds as possible for intensive care patients [21, 23]. Obstetric wards were equipped with signal lamps, non-essential furniture was removed, and one-way routes marked out. Some of the hardware for central cardiotocography monitoring was installed after a delay [5, 20]. Contact between hospital staff and infected women was kept to a minimum [26]. Some staff used the phone or other communication routes to contact women in the hospital [24].

In addition to organizational restructuring, the pandemic also led to changes in the birthing process. Several sources reported that recommendations shortening the length of Covid-19-positive women’s stays after birth had everywhere raised the number of induced labors and cesarean sections [27, 33, 42]. A New York source reported that maternity staff would perform cesarean sections on Covid-19- positive women in critical condition starting in the 24^th^ week of pregnancy, and in the 28^th^ week if the baby were in a critical condition [27]. Another report found that even a serious illness of the expectant mother in itself does not constitute an indication for c-section [24].

Postnatal care and interaction with newborns also spurred changes. As a precautionary measure, health care systems began avoiding evidence-based practices that strongly benefit mother and child. In the USA, 14% of 1,344 maternity hospitals advised against skin-to-skin contact after birth and 6.5% of hospitals forbade it [30]. Italy also advised against skin-to-skin contact at first [26]. In May 2020, four maternity hospitals in New York, where the Covid-19 rate was between 8% and 46% positive for women giving birth, transferred the infants of infected women directly to the intensive care unit [27] where they could be observed [33]. A Coxon et al. editorial claimed European hospitals were doing this too. At first, women were advised not to breastfeed [18, 33], but this advice was later mitigated [18]. Mothers in poor health were advised to pump their breastmilk into bottles [18, 26, 30] or told to wear a respiratory mask while breast-feeding, and to protect their infant from infection by following the hygiene rules [18, 24, 26]. In France, women who asked for breastfeeding support or who had other complaints (including psychological symptoms) were only offered telephone or video-call service [4]. Perrine et al. reported that women in 17.9% of American maternity hospitals seldom received support when breastfeeding [30]. Maternity staff felt burdened by the need to act contrary to evidence-based breastfeeding support [30]. Throughout the world there were reports that hospitals prematurely discharged women who had given birth [4, 24, 30, 35], even when maternity staff had reduced home visits or where postpartum visits were uncommon [4, 33, 42].

These changes and challenges directly affected the staff. A qualitative survey of fourteen midwives in Spain identified factors that posed barriers to creating a safe, respectful environment for women who had or were suspected of having Covid-19 while giving birth. They described the chaos caused at the start of the pandemic, which disrupted organization, coordination and management. They spoke of constantly changing guidelines, heavier workloads, lack of access to proper protective clothing during births, and changes in their roles as midwives. The midwives reported changes ranging from emotional support despite minimized physical contact (due to excluded companions) to dehumanization [47].

#### Exclusion of accompanying persons

Around the world, maternity caregivers began limiting the number of people at a birth. Usually only one accompanying person was permitted during clinical puerperium and to attend the birth [33]. Sometimes women in labor were allowed companionship only after dilation and partners might be allowed to stay only an hour after the birth, depending on the hospital [18, 19, 21, 24, 48, 49]. The partners of pregnant women were sometimes forbidden from attending prenatal appointments and ultrasound scans [18, 20]. Midwives across Europe were torn between continuing to offer partner-oriented care, protecting themselves from the virus, and protecting their own family members [18]. For example, a maternity hospital in France generally allowed one accompanying person during labor if that person wore a respiratory mask and gloves, but they allowed no visitors in the maternity ward (fathers could view newborn babies and mothers through a window) [4]. Italy and Japan usually excluded accompanying persons [26, 42], though hospital stays in Japan normally lasted 5-7 days [42]. Germany also implemented versions of these recommendations [17, 48]. There, women “voted with their feet” and sought out maternity hospitals that allowed an accompanying person [17], which caused some hospitals to quickly ease their restrictions soon [17]. Maternity staff advocated for allowing an accompanying person in the delivery room [19], but even when partners were permitted to attend, they sometimes had to look after their other children [20].

Maternity staff had to learn to cope with women’s anxiety and loneliness [42]. Separation was described as an important issue overall. This included the feelings maternity staff had about separating women giving birth from their families, and their attempts to compensate for that, and midwives’ own isolation from colleagues and friends, from women with whom they were prevented from having a prenatal relationship [20]. Maternity staff worried about the long-term consequences of this isolation [33]. For example, a female gynecologist was disgusted that her professional association recommended excluding partners and doulas during birth [33]. After the first peak, in the Netherlands maternity staff quickly returned to in-person meetings and partners were again allowed to attend ultrasound scans [18].

Over the course of 2020, bans on visits were eased as respiratory masks, PCR tests, and quick tests became more available [27] but practices varied. Some maternity hospitals in New York checked accompanying persons for clinical symptoms [27] and barred anyone with a ≥38°C temperature or other potential Covid-19 symptoms [21, 27, 35]. Some hospitals allowed people who had tested positive for Covid-19 a week before, did not have a temperature within the last 72 hours to attend a birth [21]. In the Netherlands, partners Covid-19 symptoms could accompany woman if they donned respiratory masks and kept their distance [18]. In Poland, Wegrzynowska et al. reported that partners with negative test results could accompany women, but the tests were expensive and difficult to procure [49].

### Main topic: subjective effects

During the pandemic crisis, maternity staff were often outside their “comfort zone” and felt that the pressures of providing normal care while coping with the pandemic placed them under strain [50]. In the midst of changing guidelines and protocols, maternity staff needs to calm upset patients and their relatives, adding additional stress [50]. Dethier & Abernathy spoke of “maintaining certainty in the most uncertain of times” [51]. Though hospitals recruited extra staff and shortening visiting hours in maternity hospitals, work load increased [50].

In cross-sectional studies carried out via an online survey with maternity staff during the Covid-19 pandemic increased anxiety and depression values predominated [7, 52–59]. Holton et al. reported that, in Australia, midwives had higher anxiety, depression and stress values than physicians and allied health staff [56]. A survey in Ireland [55] found that female professional staff were more anxious, and younger staff and administrative staff were both more anxious and more depressed. Bender et al. retrospectively compared anxiety values and job dissatisfaction during the Covid-19 pandemic to the same values before the pandemic [54]. Shah et al. compared the anxiety and depression of maternity staff to that of the general population [57]. These studies showed maternity staff had worse mental health scores than the reference. Fear of infection and concern about passing the virus on to family members increased the anxiety of maternity staff [7, 52, 53, 57, 58]. Midwives who did not work in hospitals feared passing the virus to their patients when they made home visits [28]. According to Holton et al., there was a continuing association between higher levels of anxiety, depression and stress and less clinical experience, poorer health, and more worries about Covid-19 [56]. Shah et al. found that continually changing guidelines and rapidly changing conditions caused higher anxiety and depression values [57]. In Turkey, Yörük & Güler found depression risk was 1.92 times higher among midwives than nurses [59].

A study by Uzun et al. supported the trend of higher anxiety and more depression, but when they compared physicians, midwives and nurses by age and gender, results were not significant [60]. A newspaper article from Ireland about a large online survey of midwives and nurses concluded that most participants thought pandemic harmed mental health [61]. Fear of contagion was justified because maternity staff who had trouble procuring protective clothing were infected twice as often as those with regular access to protective gear [61]. One of their greatest fears was giving the virus to family members. Several surveys and reports found that staff members isolated themselves from their families to prevent contagion [19, 53]. The British study ‘Impact of COVID-19 on the nursing and midwifery workforce’ (ICON), which was mentioned in an editorial [62], also noted how afraid nurses and midwives were of infecting family members. Of the midwives and nurses in the UK, only 1% used the online mental health forum provided by the National Health Service (NHS), perhaps because they could not muster sufficient mental capacity to reflect on their own psychological well-being [61].

A qualitative study of 14 midwives in Spain [47] who looked after women with a suspected or confirmed Covid-19 infection while they gave birth also found that the midwives were afraid they would pass the virus. They too report fear and uncertainty in situations which the midwives had to cope with suddenly on their own, about the discomfort of the protective clothing and about the lack of knowledge and support. Some of the midwives felt they could not provide the women in their care the birth experience wanted to offer them. Other midwives felt good about their work and did everything they could to create a positive, anxiety-free atmosphere [47].

Semaan et al.’s large global study show obstetricians and midwives were under more stress during the pandemic than before because staff was short (either through infection or quarantine) and their workload was higher, schedules changed frequently, and they were exhausted [33]. Kiefer et al. found that the likelihood of post-traumatic stress symptoms increased, especially in women, those who had previous traumatic experiences, and those with higher Covid-19 risk and anxiety scores [63].

Nevertheless, certain factors protect against poor mental health, including routine testing [54], protective equipment, training in managing Covid-19 [7] as well as higher resilience value [59]. Rochelson and Campbell noted that staff were less afraid of contracting Covid-19 after general testing became available in April 2020, both for standard and quick tests [5, 21].

In several studies, the negative effect of the pandemic on mental health was offset by the positive effects of the pandemic. Most of the maternity staff interviewed by Aksoy & Koçak were proud to work in the health sector [52]. Bahal et al. reported that most of them thought better of their profession and felt they were taking adequate care of mothers and newborn children [53]. Danvers & Dolan wrote that, in the face of an unknown virus, staff found working in the “familiar territory of labour and delivery” to be reassuring [19]. We also found these positive effects reflected in experiential reports. In Germany, despite changed working conditions, scarce protective equipment, and many other concerns, maternity staff felt a wave of solidarity and mutual support [28]. Fewer visitors on hospital wards fostered closer relationships between women giving birth and the person who accompanied them, and women had fewer problems breastfeeding [64]. A Letter to the Editor [65] about a small Australian pilot study that conducted semi-structured interviews with 12 physicians captured a strong sense of unit cohesiveness and reliance on collegial relationships to deal with the challenges posed by the pandemic. Staff in New York felt similarly [5, 19, 36].

Ethical dilemmas were the topic of two reviewed commentaries (one peer review [51] and one review by a journal editor [66]). Horsch et al. spoke of “moral injury” caused to staff who were forced by pandemic conditions to act against evidence, professional recommendations, or their ethical and moral values and beliefs [66]. When employees felt they were treated inhumanely it could deaden their sense of ethical and moral obligations; they might disassociate themselves as an act of self-preservation. Dethier & Abernathy described the crisis as a “heart-breaking new reality”, in which one had to work against one’s beliefs while dressed in protective clothing, e.g., separating newborns from their mothers [51]. Excluding the accompanying persons who were women’s sole support while giving birth could cause emotional overload even in experienced personnel [50]. Green et al. also discussed the maternity staff's views on excluding accompanying persons. This created more pressure on the staff to provide emotional support that had earlier been provided by family members or doulas [67]. Risk of secondary stress from exposure to the others’ traumatic also increased [50].

Finally, some health care professionals faced danger. A peer-reviewed editorial [67] from the USA reported that medical staff around the world had been victims of violent attacks because they were seen as carriers of the disease.

## Discussion

This scoping review offers a preliminary description of the effects of the Covid-19 pandemic on maternity staff in OECD countries and China. We separated our findings into two main categories. Publications reported on structural adjustments that staff had to make or challenges they had to overcome, and on subjective effects, especially on mental health. The category of structural change included five kinds of structural challenges (a-e).

Few studies focused on the experiences of maternity staff in past epidemics and pandemics [68]. The 2020 Covid-19 pandemic generated several studies on the mental health of medical staff [69–71] but these rarely included maternity staff. A few cross-sectional studies based on online surveys did include midwives but fewer still focused solely on maternity staff though they are at high risk of occupational exposure to Covid-19, especially since pregnant women are often asymptomatic for Covid-19 [19, 29] so infections may go unrecognized. Of the studies we identified, only two were based on interviews. One small qualitative pilot study interviewed Australian physicians and another interviewed only midwives who cared for infected pregnant women and women giving birth. No qualitative study has yet surveyed the whole maternity staff and solicited the views of both infected and uninfected women about care provided during the pandemic.

Maternity staff had to cope with organizational changes that created challenges like continually shifting guidelines; these findings were confirmed in all the cross-sectional online surveys. Uncertainty about the pandemic also raised fears of infection and spreading the virus. Together, these increased anxiety and stress in maternity staff. Fear of infection was not unfounded and was the greatest reason for higher anxiety [7, 52, 53, 57, 58]. A recent study showed employees in the health system were three times more likely to contract Covid-19 and pass on the virus [72]. Thus many maternity staff isolated themselves from their families [19, 53].

Though the pandemic forced hospitals to change their guidelines in response, often frequently [20, 21, 23, 30, 33, 36, 38, 48, 49], the lack of uniformity and consistency in obstetric guidelines [73] frustrated staff who also had to cope with excessive external demands.

We found that hospitals and maternity staff often created alternative communication and care options, but could not fully address them in this review. Some hospitals implemented proven telemedicine routines and systems [74]. Many of these existed and were developed further during the Covid-19 pandemic [36, 41]. Other maternity hospitals introduced digital services [45]. Other studies have focused on the effects of digital services on healthcare and the satisfaction of the female patients who use them, but we focused on the organizational and emotional effects of rapid changes in communication and care on maternity staff. We leave it to others to address in detail specific digital systems, economic barriers, and effects on quality of care. Readers interested in a scoping review about the experiences of staff and women with digital care systems in obstetrics during the Covid-19 pandemic should see Montagnoli et al. [75].

Our scoping review focused on the effects of the pandemic on maternity staff rather than on mothers and their newborns, as did Kotlar et al. They examined the direct or indirect effects of the Covid-19 pandemic on the physical, mental, economic, or social health and wellbeing of pregnant people [76]. In their scoping review they found that pregnant individuals are at a heightened risk of more severe symptoms than people who are not pregnant, that applicable guidelines varied and that severe increases in maternal health issues were reported. They also speak about rising domestic violence, decreasing prenatal visits, and the implementation of potentially harmful policies based on little evidence.

The studies we identified collected data at different time points and periods of the pandemic. The results of surveys at the peak of the first wave were different from those of surveys in summer 2020, when infections were decreasing. The focus of this scoping review was to report all published sources on the impact of the pandemic within one year. For this reason, different types of publications were included, which vary widely in terms of methodology and topics studied. Therefore, there is a large heterogeneity in the study variables and population among the included publications. Although we undertook a synthesis of the published literature, we could not compare the varied publications we identified; this is outside the purview of a scoping review. Our goal was to identify the research gap by summarizing the published literature.

A scoping review seeks to orient readers to the state of research literature, bundle research results, and communicate them. The individual studies we identified may suffer from selection bias because included participants may have been under more stress than those who did not take the survey. Non-participants may also have had less time to take part in a study.

Because the Covid-19 pandemic is still an understudied topic, factors not studied are potential confounding variables that could affect the results. But even with these potential limitations, this review has identified research gaps and can serve as a resource for future research. We suggest researchers conduct country-specific studies to systematically examine the challenges posed by pandemics and study and catalog individual coping strategies.

## Conclusion

During the Covid-19 pandemic, maternity staff coped with drastic reorganization of their work and other challenges that placed them under considerable mental strain. The effects of these stresses on maternity staff varied across healthcare systems and countries, depending on the progress of the pandemic and incidence rates. Maternity staff coped by adopting a variety of contactless or safe face-to-face communication strategies and by providing access to professional psychosocial support. Successful coping strategies were tailored to local conditions and took into account the working conditions of the maternity staff and the health of mother and child, so we recommend these temporary strategies be developed into permanent solutions that can be rapidly deployed during future pandemics.

## Supplementary Information

**Additional file 1.**

**Additional file 2.**

## Data Availability

Data sharing is not applicable to this article as no datasets were generated or analysed during the current study.

## Abbreviations

Covid-19: Coronavirus Disease 2019
SARS-CoV-2: Severe acute respiratory syndrome coronavirus 2
OECD: Organization for Economic Co-operation and Development
SARS: Severe acute respiratory syndrome
H1N1/09: H1N1-Pandemie 2009
FFP2 and N95: Safety rating systems for half-face respirators with a particle filter
