# Supplementary material for: Effects of the Covid-19 pandemic on maternity staff in 2020 – a scoping review
Source: BMC Health Serv Res. 2021 Dec 27;21:1364. doi: 10.1186/s12913-021-07377-1 (PMC8710925; doi:10.1186/s12913-021-07377-1)
Supplement: Supplementary file 2 — Additional file 2. [file 12913_2021_7377_MOESM2_ESM.docx]

Additional file 2. Search string in databases.

| Source | search string |
| --- | --- |
| CINAHL | AB (midwi* OR nurse-midwi* OR certified midwi* OR obstetric nurses OR obstetric* OR perinatal care OR maternity care) AND AB (burden OR workload OR barriers OR challenges OR safety OR stress OR mental health OR resources OR potential) AND AB (covid OR pandemic OR coronavirus) |
| MEDLINE | (AB=(midwi* OR nurse-midwi* OR certified midwi* OR obstetric nurses OR obstetric* OR perinatal care OR maternity care)) AND (AB=(burden OR workload OR barriers OR challenges OR safety OR stress OR mental health OR resources OR potential)) AND (AB=(covid OR pandemic OR coronavirus)) |
| Cochrane Library | Title Abstract Keyword (midwi* OR nurse-midwi* OR certified midwi* OR obstetric nurses OR obstetric* OR perinatal care OR maternity care) AND (burden OR workload OR barriers OR challenges OR safety OR stress OR mental health OR resources OR potential) AND (covid OR pandemic OR coronavirus) |
| PubMed | ((midwi*[Title/Abstract] OR nurse-midwi*[Title/Abstract] OR certified midwi*[Title/Abstract] OR obstetric nurses[Title/Abstract] OR obstetric*[Title/Abstract] OR perinatal care[Title/Abstract] OR maternity care[Title/Abstract]) AND (burden[Title/Abstract] OR workload[Title/Abstract] OR barriers[Title/Abstract] OR challenges[Title/Abstract] OR safety[Title/Abstract] OR stress[Title/Abstract] OR mental health[Title/Abstract] OR resources[Title/Abstract] OR potential[Title/Abstract])) AND (covid[Title/Abstract] OR pandemic[Title/Abstract] OR coronavirus[Title/Abstract]) |
